# Psychological burden and variables affected by living with a person of high risk for COVID-19 during the lockdown period in Greece

**DOI:** 10.1192/j.eurpsy.2021.1755

**Published:** 2021-08-13

**Authors:** G. Lyrakos, G. Pilafas, N. Strongylaki, D. Menti, V. Spinaris

**Affiliations:** 1 Department Of Psychiatry, General Hospital of Nikaia “Ag. Panteleimon”, Nikaia, Greece; 2 Psychology Department, Cardiff Metropolitan University at City Unity College, Athens, Greece; 3 Psychology Department, City Unity College, Athens, Greece

**Keywords:** COVID-19, stress, Psychological burden, stress resilience

## Abstract

**Introduction:**

On February 26, 2020 the Greek government established measures against the spread of COVID-19, which eventually escalated to the entire social and economic ‘lockdown’ of the state on March 23. The main message was staying home and protect the eldest who are more vulnerable to the virus.

**Objectives:**

The aim of the present study was to test the effect of living with a vulnerable person to specific psychological factors in order to be able to create future interventions for psychological well-being of the population.

**Methods:**

A convenient sample of 1,158 Greeks (280 males [24.2%] participated electronically during the ‘lockdown. A battery of questionnaires for stress resilience, acute stress, and satisfaction with life, well-being and effect on psychosocial health was used for the study. Analysis was performed with SPSS 24.

**Results:**

Individuals living in the same house with a vulnerable partner of parent were found to have statistically significant higher levels in acute stress disorder (Μ=39,4±15,4) than those living without (M=37.7±15.5) (t_1156_=2.125 p=0.03)The same happened with the effect on psychological health with the first Group having significantly higher score in the questionnaire (M=76.6±56,9) than the second group (M=69.1±55.1) (t_1156_=2.330 p=0.02). Stress resilience, satisfaction with life and well-being were not affected.

**Conclusions:**

According to our data individuals living in the same house with a vulnerable person for COVID-19 are more likely to develop acute stress and psychosocial impact. Stress reduction programs are needed in order to help this population with managing the results of the lockdown.

**Disclosure:**

No significant relationships.

